# Eupatorium Purp.—Intermittent Fever

**Published:** 1881-08

**Authors:** Clarence Willard Butler

**Affiliations:** Montclair, N. J.


### TWO CASES OF INTERMITTENT FEVER.

CLARENCE WILLARD BUTLER, M. D., MONTCLAIR, N. J.

MISS M. K. D., aged twenty-seven, dark complexion, medium height, and inclined to be stout. A pond, situated about one hundred yards from the house in which she resided, was drained off early in the spring of 1878. About noon, June 20th, 1878, I was called to see Miss D., and found that she had had a slight chill at 10 A. M., which was followed immediately by fever, in which I found her. The temperature in the axilla was 105.3°, the skin hot and dry, the pulse full, quick and rapid. Severe pain in the whole head, the face scarlet, the conjunctivæ very much congested, the head very hot (objective and subjective).

The pain in the head was throbbing in character, and the signs of active cerebral congestion of really alarming severity. Prescribed Bell. 200 in repeated doses, of a watery solution, which modified the fever and headache in about an hour. Both continued modified in degree, however, for thirty-six hours, and left her extremely weak and very "nervous."

On the 22d of June, she had another ague attack, commencing at 9 A. M., similar in character, but much modified in intensity. This case remained under my care, varying in severity and in character, till July 5th, when it presented the following condition:

The patient was very much prostrated, had lost considerable flesh; her complexion was a dirty yellow, her tongue coated continually a yellowish brown, her appetite gone, her bowels torpid, her spleen very much enlarged, and her abdomen and legs dropsical—a condition sufficiently alarming, surely. The chill appeared every day, anteponing from one-half to one and a half hours. It commenced now in the small of the back, the fingers and the toes, from which points it spreads rapidly over the body. At its height there was much shaking, with chattering of the teeth. It is accompanied with severe frontal headache of a throbbing character, with a frequent desire to evacuate the bladder, in pursuance of which she passes every five minutes a little normal urine, without pain or discomfort. She has always been, and is very thirsty with the chill, but for the last four paroxysms this thirst has been for *hot drinks*—the hotter the better; and these hot drinks (tea, hot lemonade—even hot water) are grateful, and seem to her to relieve the severity of the chill.

Fever follows the chill quickly, and runs very high. It is accompanied with thirst and aggravation of the throbbing headache. The thirst is still for hot drinks, but is less intense. The sufferings, notably the nervousness which accompanies both fever and chill, are less during the fever than during the chill.

The perspiration, which is general, is not of great amount, and is accompanied with great relief of all the sufferings.

I saw her just as the fever was leaving, and gave her *Eupatorium perpureum*, the 200th potency, in water, a dose to be taken every hour till the next chill appeared, and then stop.

July 6th. The chill came three hours later, and the entire paroxysm was less severe than at any time before since she had been sick. The thirst for hot drinks was still present, but in common with other symptoms, less marked than heretofore. To take *Sac. Lac.*

July 7th. A very slight, short paroxysm commencing later, accompanied with some thirst for *cold water*. Continue *Sac. Lac*

Although there were some malarial symptoms threatening for several days after this date, she progressed without further medication to complete health.

This consummation was aided by a temporary change of residence; but she did not make this change till there were no signs of further chills.

I was called, April 6th, 1881, to see Mrs. A. L——, a tall, awkward, very light-complexioned, Swedish woman, 30 years of age, the mother of one child, now 6 years old.

During the entire summer of 1880, in a neighboring village where she then lived, she had suffered from malaria, which commenced in April as an intermittent fever "dumb ague," etc., etc., disappearing only after frosts in autumn. With difficulty I succeeded through a most stupid interpreter in eliciting a train of symptoms for which I prescribed *Lachesis.*

April 10th, I called again and found that the *Lachesis* had made no impression whatever upon her ague. With the aid of a very intelligent interpreter, I succeeded in getting the following fair understanding of her case.

She was much debilitated, and had that anæmic appearance so often seen in the victims of malarial and quinine poisoning. During the apyrexia she is weak and unfit for exertion, but has neither pains nor aches. The chill, which commenced at 2 P. M. at first, now eight days ago, has come on every day and earlier every day till it appeared this morning at 8 o'clock. It is a severe shaking chill, commencing in the small of the back, is accompanied by some, not severe pains in the limbs and through the trunk, and lasts for about an hour. She has had thirst with the chill from the first; but with the last two chills, her thirst has been for *hot drinks* which are grateful.

The fever develops generally and quickly after the chill. It is accompanied with the same thirst (for hot drinks), with some frontal headache and gradually wears away, being followed by slight perspiration mostly in the palms of the hands and about the head.

The entire paroxysm usually lasts from two and a half to three hours.

*Eupatorium purpureum* 200 one dose, dry on the tongue, and *Sac. lac.*

April 11th. Slight paroxysm, commencing at 12 M. She had thirst but for cold drinks. *Sac. lac.*

April 12th. No appearance of chill or fever. Some perspiration during the evening. Continue *Sac. lac.*

May 12th. No return of ague to this date.

These cases are reported, not as models of treatment, since the first prescriptions were most ignoble failures in both instances, but to call attention to one or two symptoms which may prove to be valuable aids to accurate prescribing in cases of intermittent fevers.

The condition noticed in the first case of "frequent desire to pass her urine during the chill," seems worthy of remembrance in the light of the provings recorded in *Allen's Encyclopedia*, vol. IV, p. 239, symptoms 82 to 86 inclusive.

These two, with three other cases which have recovered promptly from an ague under the use of the "Trumpet weed" in my own practice, were all anteponing and four of the five were quotidian in type.

The thirst for **hot drinks* I think is true, and a valuable symptom of this drug for the reasons that it was a marked symptom in the cases above reported, noticed by the patient without suggestion by the physician, and because it was a symptom which among the last to appear before the administration of the drug, was among the first in both instances to disappear after its use.

